# Variations in the use of malaria preventive measures among pregnant women in Guinea: a secondary analysis of the 2012 and 2018 demographic and health surveys

**DOI:** 10.1186/s12936-022-04322-3

**Published:** 2022-11-01

**Authors:** Ibrahima Barry, Almamy Amara Toure, Oumar Sangho, Abdoul Habib Beavogui, Diao Cisse, Abdourahamane Diallo, Aboubacar Sidiki Magassouba, Younoussa Sylla, Lancina Doumbia, Mahamoud Sama Cherif, Alseny Yarie Camara, Fatou Diawara, Moctar Tounkara, Alexandre Delamou, Seydou Doumbia

**Affiliations:** 1 Département d’Enseignement et de Recherche en Santé Publique et Spécialités (DERSP), Université des Sciences, des Techniques et des Technologies de Bamako (USTTB), Bamako, Mali; 2 National Centre for Training and Research in Rural Health of Mafèrinyah, Forécariah, Guinea; 3 Department of Public Health, Kofi Annan University of Guinea, Conakry, Guinea; 4 Département d’Enseignement et de Recherche des Sciences Biologiques et Médicales (DERSBM), Faculté de Pharmacie (FAPH), USTTB, Bamako, Mali; 5 Department of Public Health, Faculty of Health Sciences and Techniques, Gamal Abdel Nasser University, Conakry, Guinea; 6 Service de Gynécologie, Centre Hospitalo-Universitaire Ignace Deen, Conakry, Guinea; 7 Institut National de Santé Publique Mali, Bamako, Mali; 8 African Centre of Excellence in the Prevention and Control of Communicable Diseases (CEA-PCMT), Conakry, Guinea

**Keywords:** Malaria preventive measures, Pregnant women, DHS, Guinea

## Abstract

**Background:**

Despite its effectiveness, the optimal use of the combination of insecticide-treated nets (ITN) and intermittent preventive treatment during pregnancy with sulfadoxine–pyrimethamine (IPTp-SP) remains low in malaria-endemic areas. Therefore, this study analyzed its variations and predictors in Guinea.

**Methods:**

This study was a secondary analysis of the 2012 and 2018 Guinea Demographic and Health Surveys (DHS). It included women who had given birth 3 years before each DHS, slept on ITN and took at least one dose of SP. Use was complete if a pregnant woman slept on ITNs and took SP (at least two doses in 2012; at least three doses in 2018). Moran indices were used to determine spatial autocorrelation and classification methods to identify malaria preventive measures (MPM) predictors.

**Results:**

In 2012, 60.88% of pregnant women had incomplete use of MPMs compared with 79.11% in 2018. Associated factors with incomplete MPMs in 2012 were as follows: having an indirect link with the head of household (AOR = 2.23, 95% CI 1.08–4.61) and performing at least 4 ANC visits (AOR = 0.66, 95% CI 0.44–0.99). In 2018: Living in households of 2 to 5 people (AOR = 0.54, 95% CI 0.36–0.80), have a man as the head of the household (AOR = 0.56, 95% CI 0.35–0.89), perform the first ANC in the second trimester of pregnancy (AOR = 0.74, 95% CI 0.54–0.99), perform at least 4 ANC visits (AOR = 0.47, 95% CI 0.36–0.62), have a job (AOR = 0. 67, 95% CI 0.50–0.88), give birth in a public health facility (AOR = 0.53, 95% CI 0.39–0.72) and the middle wealth quintile (AOR = 1.56, 95% CI 1.07–2.26). Analyses revealed a global autocorrelation (Moran index = 0.0009, p = 0.2349) and high–high clusters in Mamou in 2012. In 2018, autocorrelation was found (I Moran = 0.0169, p ≤ 0.05), with spatial clusters in 4 regions.

**Conclusion:**

The link with the head of household and the number of ANC visits were the main factors in MPMs. It is essential to implement strategies at the household level and health system level and monitor them to reduce inequality across regions.

**Supplementary Information:**

The online version contains supplementary material available at 10.1186/s12936-022-04322-3.

## Background

Malaria during pregnancy contributes to high maternal morbidity and mortality [1, 2]. In 2021, according to the World Health Organization (WHO), an estimated 33.8 million pregnancies occurred, of which 11.6 million (34%) were exposed to malaria infection during pregnancy [3], and west Africa had the highest prevalence (39.8%) [3]. Yet, the WHO has recommended a package of preventive measures for malaria during pregnancy, including the combined use of insecticide-treated nets (ITN) and intermittent preventive treatment during pregnancy with sulfadoxine-pyrimethamine (IPTp-SP) [1]. These two prevention measures have been proven effective [4–7]. The WHO, in its global technical strategy for malaria 2016–2030, aims for at least 80% coverage in using these measures [8]. However, challenges remain to ensure optimal use, particularly in countries with high malaria endemicity [9, 10]. For instance, in the neighbouring country of Senegal, a secondary analysis of the Demographic and Health Survey (DHS) 2013–2014 showed that 37.51% of pregnant women used this combination [11]. Similarly, according to the Global Malaria Report 2021, only 49% of pregnant women slept on ITN, and 32% received at least three doses of sulfadoxine–pyrimethamine (SP) [3]. Despite the low coverage estimates, the WHO indicated that the current levels of IPTp coverage contributed to avert an estimated 408,000 low birthweight in 2020 globally [3].

Accurate knowledge of the dynamic variations of malaria guides current preventive measures and control interventions [12]. “Demographic and Health Surveys (DHS) are nationally-representative household surveys that provide data for a wide range of monitoring and impact evaluation indicators in the areas of population, health, and nutrition” [13]. In this sense, DHS accounted at the national level, a geo-localized health data source to produce spatial risk maps [14].

In Guinea, a previous investigation showed a small proportion (23.9%) of women meeting the conditons of the complete use of malaria preventive measures (MPM) during pregnancy [15]. However, this study of nine hospital districts excluded some regions of Guinea.

The study sought the use of MPM against malaria among pregnant women who have access to them—the spatial distribution and, finally, factors that influenced its use over time in Guinea. Since the DHS is a nationwide survey, we may draw from it a sound conclusion. Therefore, the primary goal of this analysis is to identify predictors of incomplete MPM use that would help define improved strategies for increased MPM use in Guinea.

## Methods

### Study setting

Guinea is a coastal country with an area of 245,857 km^2^ located in West Africa, halfway between the Equator and the Tropic of Cancer (7° 30′ and 12° 30′ north latitude and 8° and 15° west longitude) [16]. Administratively, the country is subdivided into 8 regions (Conakry, Boké, Kindia, Mamou, Labé, Faranah, Kankan, N'zérékoré) [16]. In 2020, Guinea's population was estimated at 12,559,363, with almost 52% women [16]. Guinea has a dry season and a rainy season, each lasting 6 months [17]. The rainy season runs from May to November.

### Study design and data sources

A a secondary analysis of data from Guinea 2012 and 2018 DHS using the Guide to DHS Statistics was conducted [18]. Information on administrative region boundaries has been downloaded to the Spatial Data Repository-Boundaries (https://spatialdata.dhsprogram.com/home).

### Study population

The study population was women of childbearing age (15–49 years) who resided in ordinary households across the country during the 2012 and 2018 DHS.

### Criteria of selection

The samples considered in this analysis include data from women of childbearing age who gave birth in the three years preceding the surveys, lived in households that owned at least one ITN and had taken at least one dose of SP during pregnancy.

### Study variables

Variables in the individual recoding file (IR), which stands for female data were extracted [19].

### Dependent variable

The use of MPMs was the dependent variable. It consists of two variables: the ITN under which the women slept the night before the survey and the number of SP intakes. For the 2012 DHS, MPMs were assumed "complete" if a pregnant woman had received at least two doses of SP and slept under an ITN the night before the survey. Otherwise, the MPM was assumed "incomplete". For the 2018 DHS, MPMs were "complete" if a pregnant woman had received at least three doses of SP and slept under an ITN the night before the survey. Otherwise, they were assumed "incomplete". The difference in SP dose between 2012 and 2018 results from a change in national policy; in 2016, Guinea adopted the WHO recommendation to administer at least three doses of SP to prevent malaria during pregnancy [20].

### Independent variables

Table 1 shows all the independent variables used in the study.

**Table 1:** Independent variables of the study and their levels

No	Variables	Levels
Demography		
1	Year of DHS	2012, 2018
2	Age groups	15–18 years, 19–30 years, 31–40 years, 41–49 years
3	Administrative region	Conakry, Boké, Faranah, Kankan, Kindia, Labé, Mamou, N'Zérékoré
4	Level of education	No formal education, primary, secondary and university
5	Marital status	Single, married. Widow, divorced women and women in the separation were considered single
6	Parity	Primiparous (1 delivery), Multiparous (more than 1 deliveries)
7	Gender of head of household	Male, Female
8	Age of the head of household	16–25 years, 26–40 years, 41–60 years, 61–91 years
9	Relationships with the head of the household	We considered as a direct link any father/mother-daughter, Sister and husband-wife relationship. When the respondent was the head of the household, the link was direct. Otherwise, the link was indirect
10	Household size	The number of persons living in the household. It is divided into 2–4 people, 5–10 people, 11–38 people
11	Place of delivery	Domicile, private health structure, public health structure
12	Partner's level of education	No formal education, primary, secondary and university
13	Desire for pregnancy	Yes, no
Socio-economics		
14	Wealth Index	Poor, middle and richer. The poorest and poorer were classified as poorer, the richer and richest as richer
15	Occupation	Yes, no
Information		
16	Journal	Access, no access
17	Radio	Access, no access
18	Television	Access, no access
Health Care use		
19	Antenatal care (ANC)	Less than 4 ACNs, 4 ACNs and More
20	Time of the first ANC	The first quarter, the second quarter, the third quarter

### Data analysis

All analyses were weighted to account for the complex sampling design of the surveys [18] and proceeded with the command "*svydesign*" in R [19]. Since the missing data were few (< 5%), univariate imputation by bootstrap through *na.tools* package was used [21]. Descriptive statistics summarized demographic, socio-economic characteristics and MPMs use. The results are presented in terms of frequency and proportions. Association between the dependent and independent variables was tested using the Wald independence test for complex surveys [22]. Multivariate logistic regression were performed to identify factors associated with the incomplete use of MPMs. Indeed, a stepback procedure was used. The quality of the fit of our regression model was tested by using the Hosmer–Lemeshow test and 2 × 2 interactions between independent variables. To identify a profile of predictors of the incomplete use of MPMs, the variables of the final model of multivariate logistic regression were used in the classification and regression trees (CART), while keeping the dependent variable of multivariate logistic regression.

Regarding the spatio–temporal analysis, the proportions of incomplete use of MPM during pregnancy were filtered by DHS and administrative areas. Spatial autocorrelation was used for each DHS. The autocorrelation index, whose values vary between − 1 and 1, tests the null hypothesis that the data observed at one location are independent of data from other regions; and an index value of 0 indicates no spatial autocorrelation in the data, a negative value indicates the grouping of different values, and, a positive value means a set of similar values [23]. The existence of a local index of spatial association (LISA) was assessed using the Moran Local Index, which verifies the region's value with that of its neighbours and identifies spatial patterns. This index generates four (4) quadrants [24]: high–high (observations have higher than average values of the variable in a neighbourhood that resembles them. It is a positive spatial autocorrelation with a high index value); low–low (observations have lower than average values of the variable, in a neighbourhood that resembles them. It is a positive spatial autocorrelation with a low index value); high–low (observations have higher than average values of the variable in a neighbourhood that does not resemble them [24]. It is a negative spatial autocorrelation with a high index value); low–high (observations have lower than average variable values in a neighbourhood that does not resemble them. It is a negative spatial autocorrelation with a low index value) [24]. Lastly, Bonferroni α correction was used to check the stability of the clusters.

Statistical tests were considered significant at threshold risk α = 5%, and all analyses were performed using R version 4.02.

## Results

**Fig. 1:**
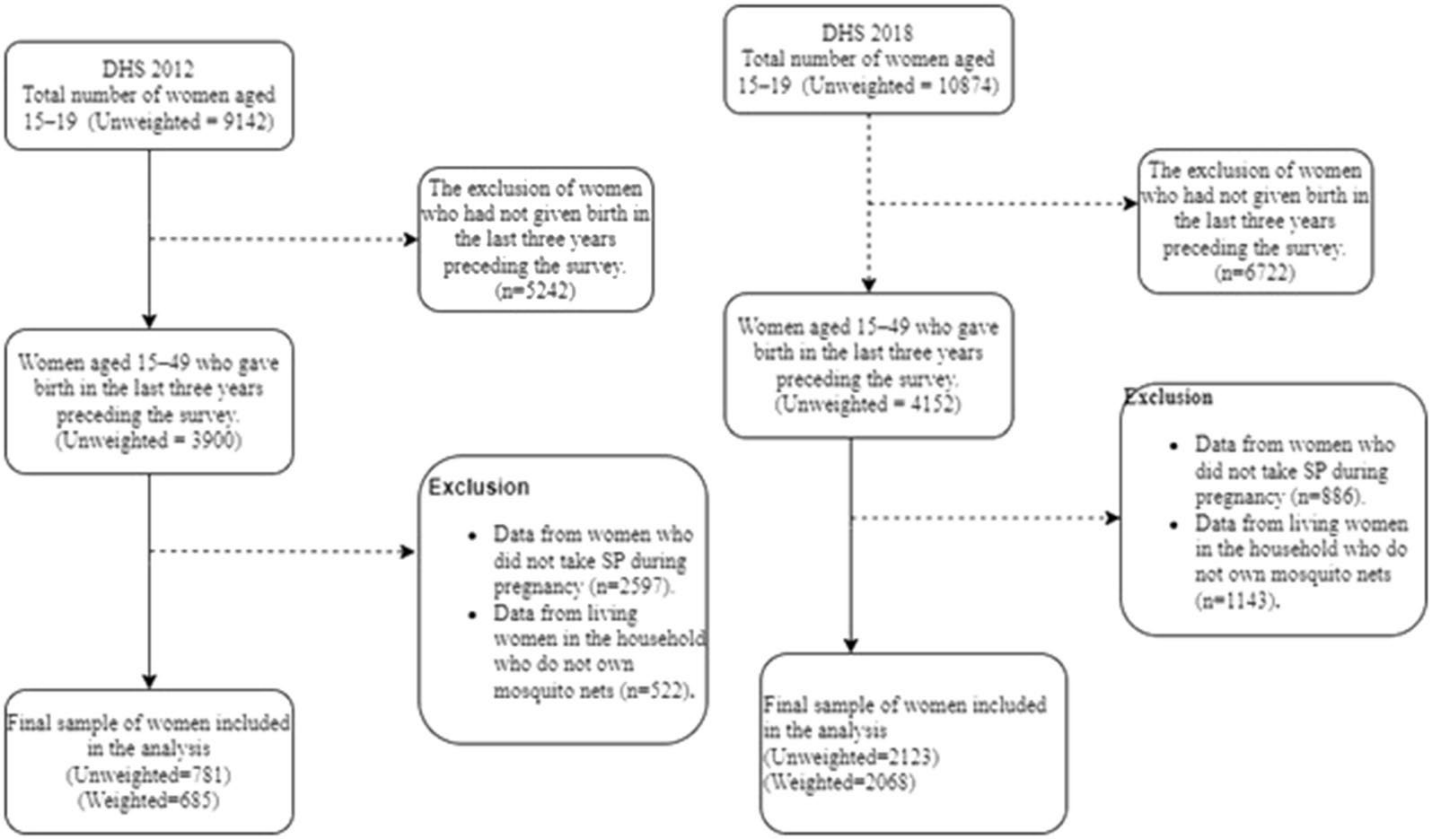
Flow inclusion diagram of DHS 2012 and 2018

Investigators interviewed 9,142 women aged 15–49 in 2012 [25] and 10,874 women aged 15–49 in 2018 [26]. The final (weighted) sample included 685 women in 2012 and 2068 women in 2018. Figure 1 showed the flow diagrams of inclusion (DHS 2012 and DHS 2018).

### Descriptive statistics

Table 2 shows a description of the study sample. The use of MPMs by pregnant women was incomplete in 60.88% of pregnant women in 2012 and 79.11% in 2018. In 2012, less than the half (45.69%) of women had completed their first ANC visit in the second trimester. In 2018, more than the half (58.41%) of women had completed their first ANC visit in the second trimester. Unlike the 2018 data, where most pregnant women (58.56%) did not perform four ANC visits, data from 2012 indicated most women (62.92%) completed at least four ANC visits. More than half of the women (52.99%) had given birth at home in 2012, while in 2018, most gave birth (54.93%) in public health facilities.

**Table 2:** Description of the study sample, DHS 2012 and 2018

Variables	DHS 2012		DHS 2018	
	N = 685^1^ N (%)		N = 2068^1^ N (%)	
Age (years)				
[16–18]	62	(9.05)	165	(7.98)
[18–30]	398	(58.10)	1225	(59.24)
[30–40]	192	(28.03)	576	(27.85)
[40–49]	33	(4.82)	102	(4.93)
Residence				
Rural	449	(65.55)	1444	(69.83)
Urban	236	(34.45)	624	(30.17)
Education				
No formal education	487	(71.09)	1468	(70.99)
Primary	92	(13.43)	262	(12.67)
Secondary	90	(13.14)	267	(12.91)
University	16	(2.34)	71	(3.43)
Household size				
[2–5]	184	(26.86)	642	(31.04)
[5–10]	339	(49.49)	1057	(51.11)
[10–38]	162	(23.65)	369	(17.84)
Journal				
access	54	(7.88)	150	(7.25)
No access	631	(92.12)	1918	(92.75)
Radio				
Access	472	(68.91)	1312	(63.44)
No access	213	(31.09)	756	(36.56)
Television				
Access	309	(45.11)	871	(42.12)
No access	376	(54.89)	1197	(57.88)
Wealth quintile				
Poor	255	(37.23)	898	(43.42)
Middle	137	(20.00)	402	(19.44)
Richer	293	(42.77)	768	(37.14)
Marital status				
Married	631	(92.12)	1891	(91.44)
Single	54	(7.88)	177	(8.56)
Working currently				
Yes	530	(77.37)	1407	(68.04)
No	155	(22,63)	661	(31,96)
Relationships with the head of household				
Direct link	568	(82.92)	1814	(87.72)
Indirect link	117	(17.08)	254	(12.28)
Gender of head of household				
Female	75	(10.95)	243	(11.75)
Male	610	(89.05)	1825	(88.25)
Age of head of household				
[16–25]	25	(3.65)	78	(3.77)
[25–40]	240	(35.04)	872	(42.16)
[40–60]	296	(43.21)	848	(41.01)
[60–91]	124	(18.10)	270	(13.06)
Parity				
Multiparous	541	(78.98)	1687	(81.58)
Primiparous	144	(21.02)	381	(18.42)
Partner's level of education				
No formal education	383	(55.91)	1447	(69.97)
Primary	132	(19.27)	151	(7.30)
Secondary	124	(18.10)	301	(14.56)
University	46	(6.72)	169	(8.17)
Moment of the first ANC				
First quarter	330	(48.18)	662	(32.01)
Second quarter	313	(45.69)	1208	(58.41)
Third quarter	42	(6.13)	198	(9.58)
Antenatal care visits				
< 4	254	(37.08)	1211	(58.56)
> = 4	431	(62.92)	857	(41.44)
Desire for pregnancy				
Yes	532	(77.66)	1744	(84.33)
No	153	(22.34)	324	(15.67)
Place of delivery				
Home	363	(52.99)	813	(39.31)
Private structure	34	(4.96)	119	(5.76)
Public structure	288	(42.04)	1136	(54.93)
Use of preventive measures				
Complete	268	(39.12)	432	(20.89)
Incomplete	417	(60.88)	1636	(79.11)

^1^Weighted

### Univariate analysis

Table 3 describes the results of the univariate analysis between the dependent and the independent variables. In 2012 and 2018, variables significantly associated with MPMs in pregnant women were as follows: the size of the household, the gender of the head of household, the age of the head of household, the period of the first ANC visits and the number of ANC visits. However, marital status (p = 0.001), head of household (p < 0.001) and parity (p = 0.009) were only significant for 2012. Newspaper exposure (p = 0.013), radio exposure (p = 0.024), wealth quintile (p = 0.036), occupation (p < 0.001) and place of delivery (p < 0.001) were only significant for 2018.

**Table 3:** Univariate analysis in the use of malaria preventive measures during pregnancy, DHS Guinea 2012 and 2018

Variables	DHS 2012			DHS 2018		
	Complete N = 268^1^ n (%)	Incomplete N = 417^1^ n (%)	p-value^2^	Complete N = 432^1^ n (%)	Incomplete N = 1,636^1^ n (%)	p-value^2^
Age (years)			0.060			0.7
[16–18]	18 (6.72)	44 (10.55)		43 (9.95)	122 (7.46)	
[18–30]	147 (54.85)	251 (60.19)		245 (56.71)	980 (59.90)	
[30–40]	90 (33.58)	102 (24.46)		123 (28.47)	453 (27.69)	
[40–49]	13 (4.85)	20 (4.80)		21 (4.86)	81 (4.95)	
Level of education			0.6			0.12
No formal education	196 (73. 13)	291 (69.78)		297 (68.75)	1171 (71.58)	
Primary	32 (11.94)	60 (14.39)		47 (10.88)	215 (13.14)	
Secondary	36 (13.43)	54 (12.95)		68 (15.74)	199 (12.16)	
University	4 (1.50)	12 (2.88)		20 (4.63)	51 (3.12)	
Household size			0.009			0.015
[2–5]	86 (32.09)	98 (23.50)		158 (36.57)	484 (29.58)	
[5–10]	134 (50.00)	205 (49.16)		215 (49.77)	842 (51.47)	
[10–38]	48 (17.91)	114 (27.34)		59 (13.66)	310 (18.95)	
Journal			0.8			0.013
Access	22 (8.21)	32 (7.67)		46 (10.65)	104 (6.36)	
No access	246 (91.79)	385 (92.33)		386 (89.35)	1532 (93. 64)	
Radio			0.3			0.024
Access	177 (66.04)	295 (70.74)		297 (68.75)	1015 (62.04)	
No access	91 (33.96)	122 (29.26)		135 (31.25)	621 (37.96)	
Television			0.5			0.080
Access	126 (47.01)	183 (43.88)		203 (46.99)	668 (40.83)	
No access	142 (52.99)	234 (56.12)		229 (53.01)	968 (59.17)	
Wealth quintile			0.3			0.036
Poorer	106 (39.55)	149 (35.73)		162 (37.50)	736 (44.99)	
Middle	47 (17. 54)	90 (21.58)		99 (22.92)	303 (18.52)	
Richer	115 (42.91)	178 (42.69)		171 (39.58)	597 (36.49)	
Marital status			0.001			0.5
Married	258 (96.27)	373 (89.45)		399 (92.36)	1492 (91.20)	
Single	10 (3.73)	44 (10.55)		33 (7.64)	144 (8.80)	
Working currently			0.4			< 0.001
Yes	212 (79.10)	318 (76.26)		327 (75.69)	1080 (66.01)	
No	56 (20.90)	99 (23.74)		105 (24.31)	556 (33.99)	
Relationship with the head of household			< 0.001			0.075
Direct link	245 (91.42)	323 (77.46)		390 (90.28)	1 424 (87.04)	
Indirect link	23 (8.58)	94 (22.54)		42 (9. 72)	212 (12.96)	
Gender of head of household			0.049			0.007
Female	20 (7.46)	55 (13.19)		34 (7.87)	209 (12.78)	
Male	248 (92.54)	362 (86.81)		398 (92.13)	1427 (87.22)	
Age of head of household			0.027			0.036
[16–25]	15 (5.60)	10 (2.40)		14 (3.24)	64 (3.91)	
[25–40]	102 (38.06)	138 (33.09)		194 (44.91)	678 (41.44)	
[40–60]	116 (43.28)	180 (43.17)		185 (42,82)	663 (40.53)	
[60–91]	35 (13.06)	89 (21.34)		39 (9.03)	231 (14.12)	
Parity			0.009			0.2
Multiparous	225 (83.96)	316 (75.78)		342 (79.17)	1345 (82.21)	
Primiparous	43 (16.04)	101 (24.22)		90 (20.83)	291 (17.79)	
Partner's level of education			0.11			0.3
No formal education	146 (54.48)	237 (56.83)		281 (65.05)	1166 (71.27)	
Primary	43 (16.05)	89 (21.34)		32 (7.41)	119 (7.27)	
Secondary	62 (23.13)	62 (14.87)		76 (17.59)	225 (13.75)	
University	17 (6.34)	29 (6.96)		43 (9.95)	126 (7.70)	
Moment of the 1st CPN			0.001			< 0.001
First quarter	127 (47.39)	203 (48.68)		140 (32.41)	522 (31.91)	
Second quarter	134 (50.00)	179 (42.93)		273 (63.19)	935 (57.15)	
Third quarter	7 (2.61)	35 (8.39)		19 (4.40)	179 (10.94)	
CPN name			0.007			< 0.001
< 4	81 (30.22)	173 (41.49)		185 (42.82)	1026 (62.71)	
> = 4	187 (69.78)	244 (58.51)		247 (57.18)	610 (37.29)	
Desire for pregnancy			0.7			0.3
Yes	206 (76.87)	326 (78.18)		373 (86.34)	1371 (83.80)	
Not	62 (23.13)	91 (21.82)		59 (13.66)	265 (16.20)	
Place of delivery			0.10			< 0.001
Home	125 (46.64)	238 (57.07)		116 (26.85)	697 (42.60)	
Private structure	17 (6. 34)	17 (4.08)		26 (6.02)	93 (5.68)	
Public structure	126 (47.02)	162 (38.85)		290 (67.13)	846 (51.71)	

^1^Weighted

^2^Wald independence test for complex survey samples

### Multivariate logistic regression

Table 4 shows the results of the multivariate logistic regression. In 2012, incomplete use of MPM was 1.99 times higher among single women than among married women (AOR = 1.99, 95% CI 1.01–3.94). Similarly, having an indirect link with the head of household increased incomplete use of MPM by 2.23 times (AOR = 2. 23, 95% CI 1.08–4.61). Women who attended at least four ANC visits were less likely to have incomplete MPM than those who attended less than four ANC visits (AOR 0.66, 95% CI 0.44–0.99). In 2018, women living in households of more than ten people were two times more likely to have incomplete use of MPMs than those living in households of two to five people (AOR = 0.54, 95% CI 0.36–0.80). Similarly, women living in households of between six and 10 people were 25% less likely to have incomplete MPM, but that association was not significant. Incomplete use of MPMs by pregnant women was significantly reduced by two times when the head of the household was male (AOR = 0.56, 95% CI 0.35–0.89). Performing the first ANC in the second quarter of pregnancy significantly reduced incomplete use of MPM by 1.35 times (AOR = 0.74, 95% CI 0.54–0.99). As in 2012, women who did not attend four ANC visits were significantly two times more likely to have incomplete use of MPMs than their counterparts who attended at least four ANCs (AOR = 0.47, 95% CI 0.36–0.62).) Not having access to newspapers increased pregnant women’s chances of incomplete MPMs by 1.54 times (AOR = 1.54, 95%; CI 1.02–2.34). Pregnant women who were not working were 1.5 times more likely to have incomplete use of MPM than those who were working (AOR = 0.67, 95% CI 0.50–0.88). In addition, women who gave birth at home were significantly two times more likely to have incomplete MPMs during their pregnancy than those who gave birth in a public health facility (AOR = 0.53, 95% CI 0.39–0.72). Moreover, incomplete use of MPM was significantly 1.56 times higher among women in wealthy households than among those in households in the middle wealth quintile (AOR = 1.56, 95% CI 1.07–2.26). Similarly, women in the poorer quintile were 1.23 more likely to have incomplete use of MPM than the middle quintiles, but the relation was not significant.

**Table 4:** Multivariate analysis in the use of malaria prevention measures during pregnancy, DHS data 2012 and 2018, Guinea

Variables	DHS 2012			DHS 2018		
	AOR	CI 95%	p-value	AOR	CI 95%	p-value
Household size						
[10–38]	–	–		–	–	
[2–5]	0.60	0.34, 1.06	0.077	0.54	0.36, 0.80	0.003
[5–10]	0.73	0.46, 1.16	0.2	0.75	0.53, 1.06	0.10
Marital status						
Married	–	–				
Single	1.99	1.01, 3.94	0.047			
Relationship with the head of household						
Direct link	–	–				
Indirect link	2.23	1.08, 4.61	0.030			
Gender of head of household						
Female	–	–		–	–	
Male	0.62	0.32, 1.22	0.2	0.56	0.35, 0.89	0.015
Age of the head of household						
[25–40]	–	–		–	–	
[16–25]	0.47	0.18, 1.18	0.11	1.20	0.60, 2.40	0.6
[40–60]	0.97	0.62, 1.51	0.9	0.91	0.70, 1.18	0.5
[60–91]	0.95	0.54, 1.69	0.9	1.35	0.87, 2.09	0.2
Moment of the first ANC						
First quarter	–	–		–	–	
Second quarter	0.75	0.50, 1.13	0.2	0.74	0.54, 0.99	0.045
Third quarter	2.32	0.95, 5.65	0.064	1.56	0.93, 2.62	0.094
ANC visits						
< 4	–	–		–	–	
> = 4	0.66	0.44, 0.99	0.043	0.47	0.36, 0.62	< 0.001
Parity						
Multipare	–	–				
Primiparous	1.31	0.83, 2.06	0.2			
Journal						
Access				–	–	
No access				1.54	1.02, 2.34	0.042
Radio						
Access				–	–	
No access				1.23	0.93, 1.63	0.15
Occupation						
Not				–	–	
Yes				0.67	0.50, 0.88	0.005
Place of delivery						
Home				–	–	
Private structure				0.74	0.40, 1.40	0.4
Public structure				0.53	0.39, 0.72	< 0.001
Wealth Index						
Middle				–	–	
Poorer				1.23	0.87, 1.74	0.2
Richer				1.56	1.07, 2.26	0.021

AOR: adjusted odds ratio; CI: confidence intervalle

### Classification and regression trees

**Fig. 2:**
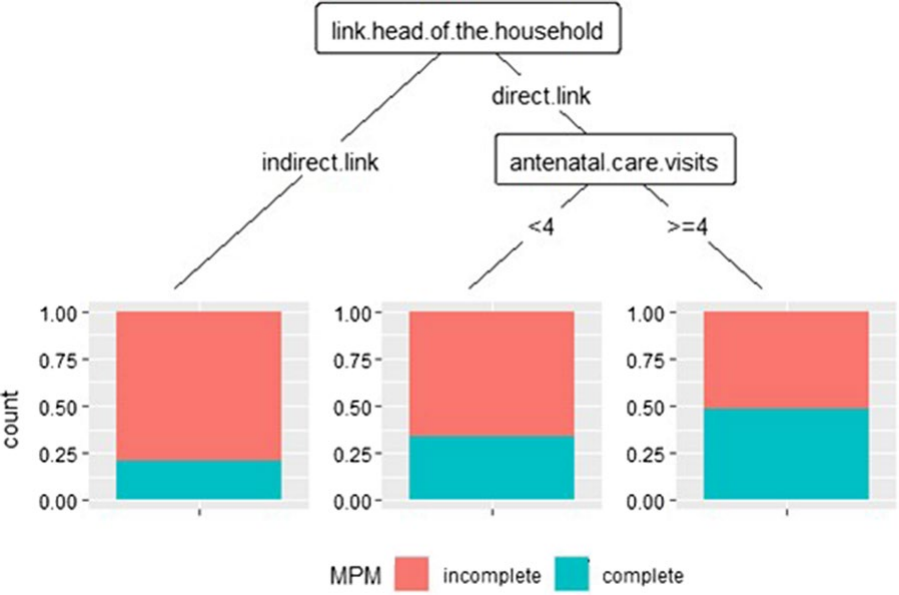
Profiles of predictors in the use of MPM with CART. DHS 2012

**Fig. 3:**
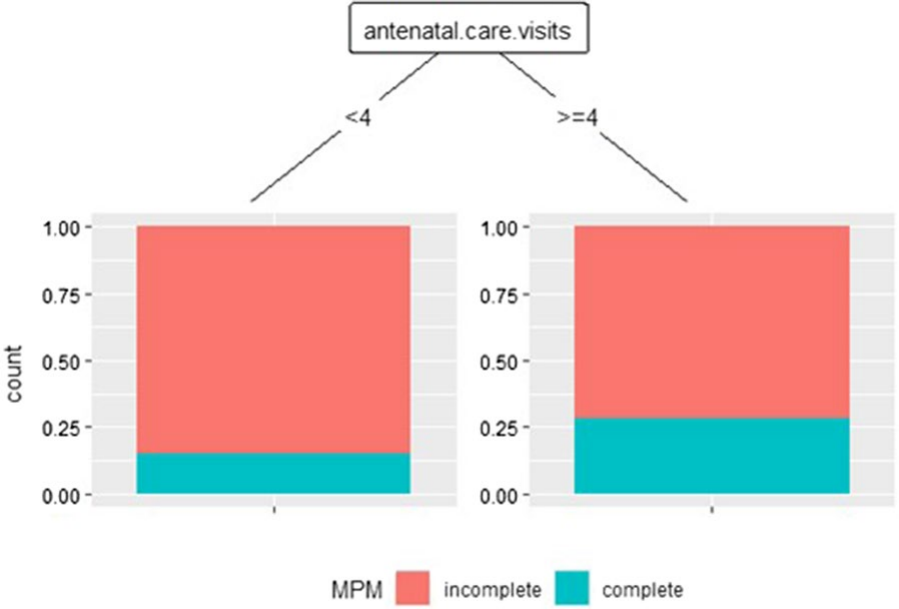
Profiles of predictors in the use of MPM with CART. DHS 2018

Figure 2 shows that the link with the head of household makes it possible to distinguish three main clusters using MPMs according to the direct link (d-link) or indirect link and the number of ANC visits attended. Cluster 3, women who had a direct relationship with the head of the household and had completed less than four ANCvisits. Cluster 4, women who had a direct relationship with the head of the household and had completed at least 4 ANC visits. Cluster 5, women had an indirect link with the head of the household. The risk of incomplete use of MPMs during pregnancy was higher (80%) when women had an indirect link to their head of household. Conversely, when this link was direct and the women had at least four ANC visits, the risk of incomplete MPMs was reduced by half (50%). Figure 3 shows that the risk of incomplete use of MPMs during pregnancy was higher among women who had not attended at least four ANC visits.

### Spatial analysis

**Fig. 4:**
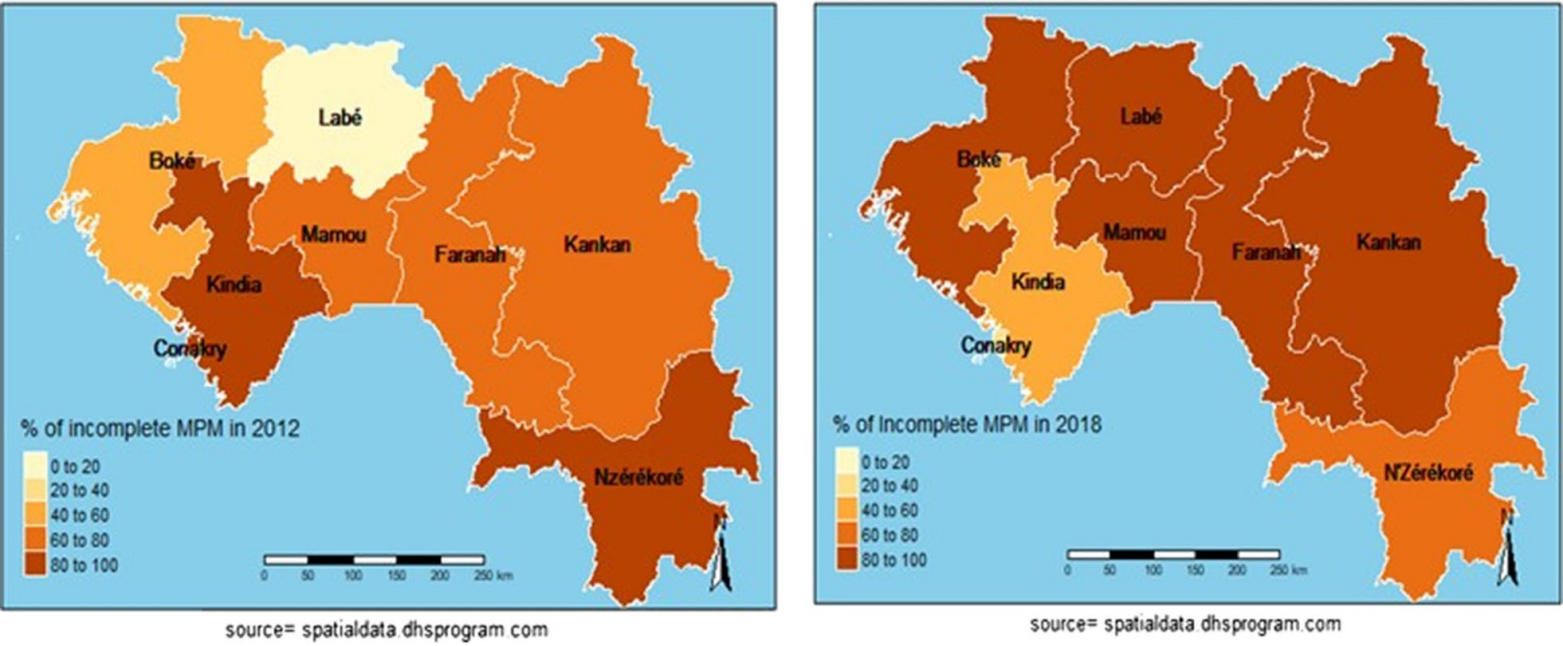
Incomplete use of MPM among pregnant women. DHS 2012/DHS 2018

**Fig. 5:**
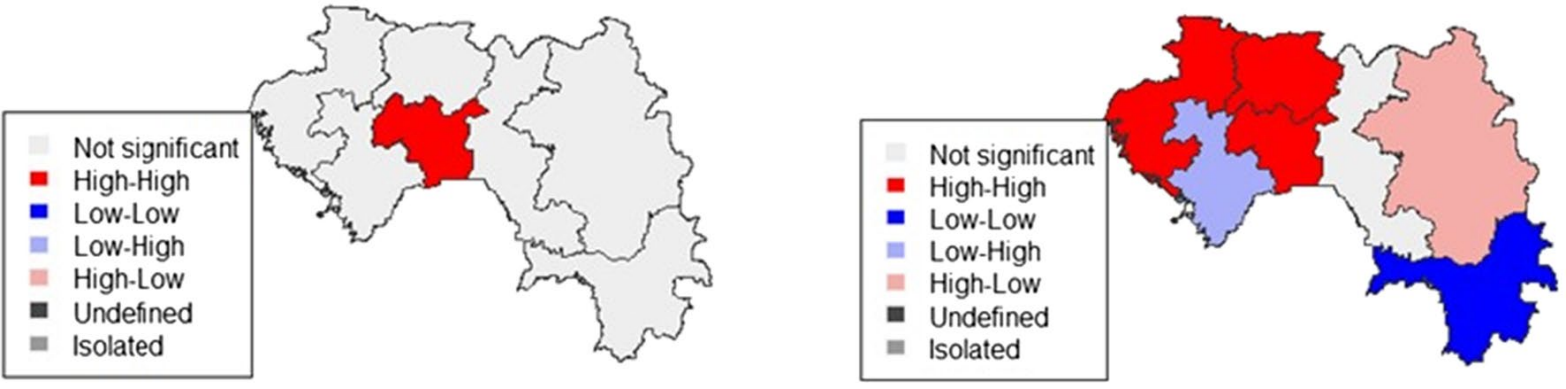
Moran's local index map of incomplete use of MPM among pregnant women, DHS 2012/2018

**Fig. 6:**
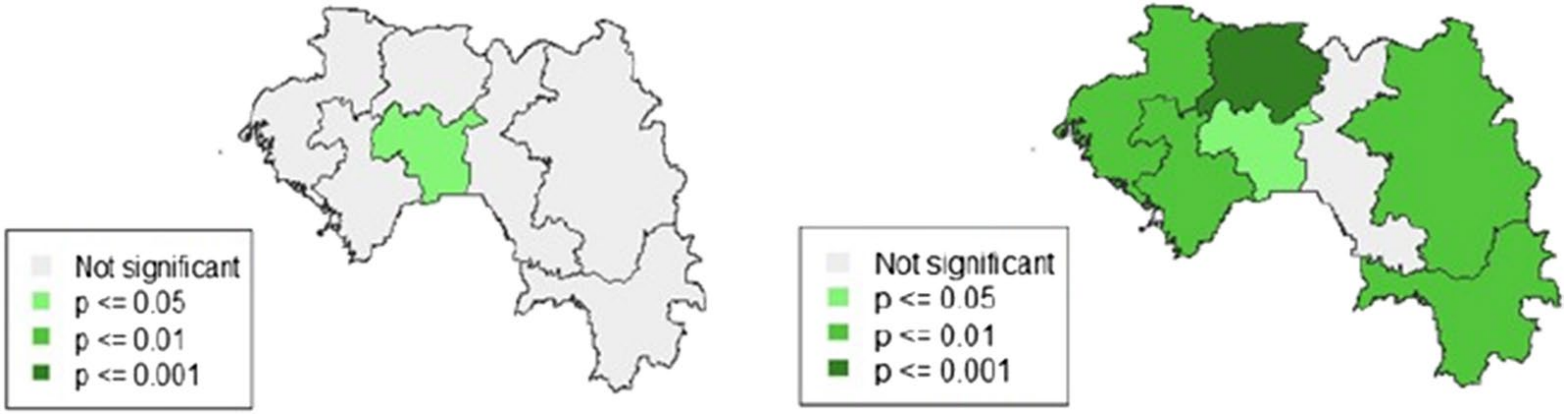
Moran's local significance of incomplete use of MPM among pregnant women. DHS 2012/2018

In 2012, Fig. 4 shows that the regions of Kindia, N'zérékoré and Conakry have the highest proportions of incomplete use of MPMs by pregnant women. However, Labé had the lowest ones. The global Moran index was 0.0009 in 2012, p = 0.2349 (Table 5), indicating a non-significant positive spatial autocorrelation. The Moran local index identified spatial clusters with the high–high model in the region of Mamou (Fig. 5). In 2018, the highest proportion of incomplete use of MPMs was found in the regions of Boké, Mamou, Labé, Faranah, and Kankan (Fig. 4). The use of MPMs was better in N'zérékoré, Kindia, and Conakry (Fig. 4). Significant positive spatial autocorrelation was found (overall Moran index in 2018 = 0.0169; p ≤ 0.05) (Table 5). The main high–high pattern was spotted in Boké, Mamou, and Labé (Figs. 5 and 6). Low–low clusters were identified in the region of N'zérékoré (Figs. 5 and 6). Lastly, low–high and high–low clusters were found in the regions of Kindia and Kankan (Figs. 5 and 6).

**Table 5:** Measurement of spatial autocorrelation of incomplete use of MPM during pregnancy

Parameters	Moran's overall index	p-value
DHS 2012	0.0009	0.235
DHS 2018	0.0169	≤ 0.05

## Discussion

Investments continue to grow in the fight against malaria in Guinea. However, the coverage of MPMs remains low. The proportion of incomplete MPMs between the 2012 and 2018 DHSs was assessed. The occurrence of Ebola virus disease (EVD) in 2014 could be one of the most significant factors influencing this state of fact. EVD has disrupted health care delivery and created mistrust of health facilities [27]. However, we noticed that the use of MPMs by pregnant women is low in countries with high malaria endemicity [11, 28, 29]. Those findings could result from the fact that strategies are more focused on the distribution of preventive measures than the importance of their use [30]. Analyses revealed that insufficient ANC visits strongly predict incomplete MPMs, as confirmed in other settings [10, 11]. Hence, it is crucial to reinforce strategies that improve attendance of ANC visits.

Data from the 2012 DHS revealed that marital status and relationship with the head of household were significantly associated with MPMs use. Indeed, married women were more likely to use MPMs during pregnancy than single women, who require special attention because they can remain hidden and resort to self-medication [31]. In fact, in Nigeria, women reported needing the support or consent of their husbands before going to an ANC or taking the medication [28]. The existence of a direct link between the pregnant woman and the head of the household halved the risk of incomplete use of MPMs. In Guinea context, women often have to rely on the head of the household to make decisions about access to ITN or the use of the household income to pay for antenatal care services [32]. It is crucial to increase the awareness of the heads of the household to increase the complete use of MPMs.

In contrast, data from the 2018 DHS showed that the timing of the 1st ANC, household size, gender of the head of household, access to newspapers, occupation, place of delivery and wealth quintile were the predictors of the complete use of MPMs during pregnancy. The probability of incomplete use of MPMs was significantly higher when the woman performed her first ANC in the 1st quarter of pregnancy instead of the second trimester. A similar finding was reported in Uganda [10]. Indeed, the WHO recommends the administration of IPTp-SP during ANC from the second trimester of pregnancy [1]. Living in households of 10–38 people were significantly more likely to have incomplete use of MPMs during pregnancy than those living in households of less than six. Family size is negatively associated with the use of ITN at the individual level [33]. Indeed, according to data from the 2018 DHS, among households that have at least one ITN, 27% do not have enough of them to protect all household members [20]. Incomplete use of MPMs during pregnancy was high regardless of the sex of the head of the household, but decreased when the head of the household was male. Previous studies support this finding, as male-headed households have more financial resources and better access to health information [10, 33, 34]. Furthermore, almost all women who were not working had incomplete use of MPMs during pregnancy. A paid job gives women more chances to use complete MPMs [32].

No access to newspapers significantly increase the incomplete use of MPMs during pregnancy, suggesting a strengthening of awareness messages via the media for better impact. Moreover, results showed that women who gave birth at home had higher incomplete use of MPMs during pregnancy than those who gave birth at public health centres. Indeed, attendance at public health facilities for antenatal care influences the uptake of optimal doses of SP by pregnant women [34, 35]. Also, women in wealthy quintile households were significantly more likely to have incomplete use of MPMs than those in the middle quintile households. This result is consistent with that found by other authors [10, 11]. Diallo et al. suggest that in wealthy households, women may use other means of protection such as fans or air conditioners [15].

Analyses found that the Conakry, Kindia, and N'Zérokoré regions had higher proportions of incomplete MPM in 2012, though higher rates of completion of MPMs were seen in 2018. During the Ebola epidemic, authorities paid much attention to those areas [36]. Many changes occurred (renovation of health centres, recruitment of new health worker and their capacity building, increase in the share of state health financing from 3 to 8% etc.) to face the system failure caused by the outbreak. From 2012 to 2018, regional disparities were found in the use of MPM. In 2012, high–high spatial clusters were observed only in the region of Mamou, indicating a similar high proportion of incomplete usage in the neighbouring areas. In 2018, high–high space clusters were found in Boké, Mamou, and Labé. Also, the presence of high–low clusters in the region of Kankan showed a high proportion of incomplete use of these measures in this area, unlike in neighbouring regions. Regions of Kindia (low–high) and N'zérékoré (low–low) performed well in terms of MPM. These results highlight the need to strengthen malaria prevention in pregnant women.

## Strengths and limitations

This is the first study on the combined use of ITN and SP (MPMs) during pregnancy in Guinea to include spatial analysis. Despite this, there have some limitations. In fact, during DHS, information about taking SP was based solely on women's memory. Nevertheless, this potential recall bias was minimized by limiting the analysis to births in the last 3 years before the surveys. Also, the use of ITN during pregnancy is based on sleeping under ITN the night before the survey. Yet, it is possible that the women used ITN during their pregnancies but did not use it the night before the survey. Additionally, study did not examine all factors that might influence the complete use of malaria preventive measures during pregnancy, such as adverse effects of SP, number and quality of mosquito nets per household. Lastly, a socio-anthropological study could also help better understand the low coverage of MPM among pregnant women.

## Conclusion

This study underscores the non-decrease in a high proportion of MPM measures during pregnancy between the two DHS (2012 and 2018) in Guinea. The link with the head of household (DHS 2012) and the number of ANCs (DHS 2018) were the main factors in malaria preventive measures. Significant disparities were found in MPM use through the two DHS (2012 and 2018) and between neighbouring regions. It is essential to implement strategies at the household level (by paying attention to the link between pregnant women and the head of household) and health system level (by strengthening ANC visits) and monitor them to reduce inequality across regions.

## Supplementary Information

**Additional file 1.** The R script used for the analysis is included in the manuscript.

## Data Availability

The datasets analysed during the study were obtained with permission from ICF International and are available at https://dhsprogram.com/data/dataset/Guinea_Standard-DHS_2018.cfm?flag=0. The R script used for the analysis is included in the manuscript (Additional file 1).

## Abbreviations

ANC: Antenatal care visit
AOR: Adjusted odds ratio
CART: Classification and regression trees
DHS: Demographic and health survey
EVD: Ebola virus disease
IPT_p_-SP: Intermittent preventive treatment of malaria in pregnancy using sulfadoxine–pyrimethamine
ITN: Insecticide-treated mosquito nets
MPMs: Malaria preventive measures
SP: Sulfadoxine–pyrimethamine
WHO: World Health Organization
